# Antibiotic resistance in Campylobacter and other diarrheal pathogens isolated from US military personnel deployed to Thailand in 2002–2004: a case–control study

**DOI:** 10.1186/s40794-017-0056-y

**Published:** 2017-07-05

**Authors:** Carl J. Mason, Siriporn Sornsakrin, Jessica C. Seidman, Apichai Srijan, Oralak Serichantalergs, Nucharee Thongsen, Michael W. Ellis, Viseth Ngauy, Brett E. Swierczewski, Ladaporn Bodhidatta

**Affiliations:** 1 Armed Forces Research Institute of Medical Sciences, Bangkok, 10400 Thailand; 2 Fogarty International Center, National Institutes of Health, Bethesda, MD 20892 USA; 3 Department of Medicine, University of Toledo College of Medicine and Life Sciences, Toledo, OH 43606 USA; 4 Department of Medicine, Tripler Army Medical Center, Honolulu, HI 96859 USA

**Keywords:** *Campylobacter*, Traveler’s diarrhea, Military personnel, Cobra Gold, Thailand, Antibiotic resistance

## Abstract

**Background:**

Campylobacter continues to be an important cause of diarrheal disease worldwide and a leading cause in Southeast Asia. Studies of US soldiers and marines deployed to Thailand for a 2 to 3 week field exercise provide a unique population in which to study traveler’s diarrhea.

**Methods:**

A case–control study of 217 deployed military personnel was conducted from 2002 through 2004. Of these, 155 subjects who presented to a field medical unit with acute diarrhea were enrolled as cases. These subjects referred an additional 62 diarrhea-free colleagues who served as controls. Frequencies of isolation of *Campylobacter* spp. and other enteric pathogens were compared in cases and controls, and antibiotic resistance of isolates was described.

**Results:**

Of the 155 subjects with diarrhea, *Campylobacter* spp. was the most commonly identified pathogen, found in 54 (35%) of the subjects, followed by non-typhoidal *Salmonella* species found in 36 (23%) subjects. Of the 57 separate *C. jejuni and C. coli* isolates from these individuals, 51 (89%) were resistant to ciprofloxacin by the disc diffusion method. Nearly one-third of the *Campylobacter* species were resistant to ampicillin and trimethoprim-sulfamethoxazole. Resistance to azithromycin remained low at 2% (*n* = 1).

**Conclusions:**

The significant morbidity and marked fluoroquinolone resistance associated with *Campylobacter* infections in Thailand are important considerations for clinicians providing counseling on appropriate antibacterial regimens for civilian and military travelers.

## Background

Acute infectious diarrhea poses a significant threat to international travelers and deployed military personnel particularly during travel in tropical regions where the risk of acquiring diarrhea is estimated to be 30–50% [1]. Although there are local and regional variations in etiology, the majority of traveler’s diarrhea is caused by bacterial pathogens [2]. Globally, enterotoxigenic *Escherichia coli* (ETEC) are most frequently isolated from travelers with diarrhea; however other organisms such as *Campylobacter* and *Salmonella* contribute significantly to the burden of traveler’s diarrhea, especially in South East Asia and Thailand [3–7].

*Campylobacter* causes more severe and debilitating illness than most other causes of travelers’ diarrhea [6, 8, 9]. The clinical presentation of *Campylobacter* enteritis can range from a mild, watery diarrhea to a dysenteric disease with fecal leukocytes and frank blood [10]. Some strains are capable of eliciting a post-infectious polyneuropathy, Guillain-Barre Syndrome [11]. In both non-immune and indigenous populations, *Campylobacter* resistance to standard treatment regimens has increased. Resistance of *Campylobacter* to ciprofloxacin was reported to be above 80% in Thailand in the 1990’s [12].

Deployed US military personnel are a unique study population. Cobra Gold is a country-wide, multilateral military training exercise held annually in Thailand. It is well suited for diarrheal disease studies due to the availability of good laboratory support and a large, identifiable group of travelers with predictable schedules who can be identified and followed over a one month period [5]. Previous studies in US soldiers deployed to Thailand for this exercise showed *Campylobacter* was the leading cause of diarrhea [7–9, 13–15]. Here we report on the monitoring activities conducted during Cobra Gold 2002–2004 exercises. Periodic disease surveillance is critical for determining year-to-year variation in pathogen-specific etiologies, identifying the circulating strain serotypes, and monitoring drug resistance. Similar to a previous study [9], we have included a diarrhea-free control group in order to assess the etiologic importance of isolated stool pathogens including *Campylobacter*, *Shigella*, *Salmonella*, *Vibrio*, *Aeromonas*, *Plesiomonas* and diarrheagenic *E. coli*.

## Methods

This study obtained scientific and ethical approval from the Walter Reed Army Institute of Research Human Use Review Committee. The study population consisted of US soldiers, sailors, and marines who participated in the Operation Cobra Gold 2002–2004. Field laboratories and data collection teams were established at each study site (Sa Kaeo in 2002; Prachuap Khirikhan in 2003; Chonburi in 2004 and Nakornratchasima in 2004). The purpose and availability of the study was explained to the staff of the units at the study site. Posters were placed throughout the study site. Personnel presenting to the field medical unit at the study site with acute diarrhea were recruited as cases. Acute diarrhea was defined as three or more loose stools in a 24-h period. Each control subject was an asymptomatic colleague referred by a case for potential participation. Exclusion criteria for control subjects included diarrhea, fevers, or use of antimicrobials in the prior week. The study was explained by team members and each subject provided signed informed consent. Names and social security numbers were used to ensure accurate reporting of findings to the military medical staff treating patients, but were not used during laboratory or data analysis.

Each subject provided a stool sample. Stool specimens were cultured on-site. Stool specimens were processed within 4 h. Isolate identification and antibiotic susceptibility testing was done at the Armed Forces Research Institute of the Medical Sciences in Bangkok. Stool specimens were inoculated into MacConkey (MAC), Hektoen Enteric, thiosulfate-citrate-bile salts sucrose, modified semi-solid Rappaport-Vassiliadis (MSRV) media before and after enrichment in selenite F, alkaline peptone water and buffer peptone water broths. All agar media and broths were incubated at 37 °C for 18–24 h except MSRV media which was incubated at 42 °C for 24 h. Primary and secondary plating for *Campylobacte*r was also performed. Six to 8 drops of fecal suspension were applied to 0.65 μm sterile cellulose acetate membrane filters (Millipore, USA) centrally placed on the surface of Brucella agar (Difco, USA) with 5% sheep blood plates. After a 30-min incubation at room temperature to allow filtration, membranes were removed and the plates incubated at 37 °C under microaerobic conditions (generated by a Gas Pak) for 48 h. In addition, approximately 0.5 ml of the remaining fecal suspension was placed in Preston selective enrichment broth and incubated for 48 h at 37 °C under microaerobic conditions prior to carrying out the membrane filtration technique described above.

*Shigella,* non-typhoidal *Salmonella, Vibrio, Aeromonas* and *Plesiomonas shigelloides* were identified by standard biochemical methods [16]. *Shigella* and *Vibrio* spp. were serotyped using Denka-Seiken antisera. *Salmonella* spp. was serogrouped using Serotest antiserum (S&A Reagents Lab, Thailand). Up to five lactose fermenting and five non-lactose fermenting colonies of *E. coli* (as identified on MAC agar and confirmed by biochemical typing) were saved on Dorset egg yolk media slant. Diarrheagenic *E. coli* isolates were identified by hybridization with specific digoxigenin-labeled polynucleotide DNA probes: heat-labile toxin (LT), and heat-stable toxins STIa (STp) and STIb (STh) probes for Enterotoxigenic *E. coli* (ETEC) [17]; Bundle forming protein A (BfpA) and effacing and attaching (EAE) probes for Enteropathogenic *E. coli* (EPEC) [18, 19]; and pCVD432 probe for Enteroaggregative *E.coli* (EAEC) [20]. Colonization factor antigens for ETEC isolates were identified by dot immunoblot assay [21–23].

Giardia/cryptosporidium detection was performed using commercial ProspectT® Microplate assay (Alexon-Trend, USA). Stool samples were tested for rotavirus, adenovirus and astrovirus by a commercial ELISA test (R-Biopharm AG, Germany).

All isolates of *Shigella*, *E. coli*, *Campylobacter*, *Vibrio, Aeromonas, P. shigelloides,* and *Salmonella* were tested for antimicrobial susceptibility to ampicillin, azithromycin, ciprofloxacin, nalidixic acid, trimethoprim/sulfamethoxazole and tetracycline by the disc diffusion method as recommended by NCCLS [24, 25] using commercially prepared antibiotic discs (BD Diagnostic Systems, Sparks, MD, USA). *Campylobacter* isolates were tested for susceptibility to antimicrobial drugs using the disk diffusion assay with modifications. Because no standardized interpretive criteria existed for Campylobacter spp., the inhibition zone diameters were measured and interpreted following the disk manufacturer’s instructions and compared against the Clinical and Laboratory Standards Institute (formerly NCCLS) standard guidelines for aerobic gram-negative bacilli to interpret the results as susceptible, intermediate, or resistant.

Risk Questionnaires regarding risk factors were completed by the study subjects. In all years, subjects were surveyed about travel history (including previous travel to Cobra Gold exercises, Thailand and South East Asia) and recent illness symptoms, and medication use. Risk Questionnaires from 2002 and 2003 asked about sources of food consumed (on-base: military dining facility, civilian establishment; off-base: street vendors, traditional Thai restaurants, and non-traditional restaurants (e.g. Pizza Hut)) and exposure to specific foods and drinks on- and off-base in the preceding five days.

Data were entered into an Epi-Info version 3.3 database. Statistical analysis was performed using IBM SPSS Statistics version 23, computed for chi-square tests/Fisher’s exact tests, odds ratio and 95% confidence interval (CI) by two-way tables and multivariate logistic regression and considered statistically significant using a two-tailed *p*-value <0.05.

## Results

### Study population

Over the three enrollment years, a total of 159 US soldiers or marines with acute diarrhea participated in the study as cases while only 66 of their asymptomatic colleagues participated as control subjects. Eight subjects (4 diarrhea cases and 4 non-diarrhea controls) had incomplete data and were excluded from analysis. The overall enrollment numbers were consistent from year to year and site to site. Nearly all volunteers were male, reflecting the demographics of the surveyed population, and most participants were junior in rank (Table 1).

**Table 1:** Numbers, locations and demographic data of diarrhea cases and controls during Operation Cobra Gold, 2002–2004

Description	Case	Control	Total
Year			
2002	52	21	73
2003	48	26	74
2004	55	15	70
Location			
Sa Kaeo, 2002	52	21	73
Prachuap Khirikhan, 2003	48	26	74
Chonburi, 2004	34	7	41
Nakornratchasima, 2004	21	8	29
Sex			
Male	150	60	210
Female	5	2	7
Grade Group			
Junior Enlisted (E1–E4)	82	29	111
Noncommissioned Officer (E5–E9)	47	23	70
Officer (O1 - O9)	19	8	27
Other	7	2	9
Total	155	62	217

### Organisms isolated

Of 155 cases and 62 controls, *C. jejuni*, ETEC and *P. shigelloides* were isolated significantly more frequently from cases than controls (*p* < 0.01) (Table 2). Among the 54 cases positive for *Campylobacter* spp., *C. jejuni* was isolated from 45 (83%) and *C. coli* was isolated from 10 (20%); one case was positive for both species. Although isolation of diarrheagenic *E. coli* was not significantly associated with case status, subgroup analysis revealed 17 of the 18 ETEC isolations were from cases (*p* = 0.03). All of the 17 ETEC isolated from diarrheal cases were ST-ETEC and 1 LTST-ETEC was from non-diarrhea control. The colonization factors (CFs) of ST-ETEC were characterized as CS6 (35%), CS2,3 (6%), PCFO166 (6%) and unidentified CF (52%). EPEC and EAEC isolation was similar between cases and controls. The frequency of non-typhoidal *Salmonella* isolation was not significantly different between cases and controls.

**Table 2:** Percentage of enteric pathogens isolated from diarrhea cases and controls during Operation Cobra Gold, 2002–2004

Pathogen	Cases *n* (%) (*N* = 155)		Controls *n* (%) (*N* = 62)		Odds Ratio (95% CI)		*p*-value^a^
*Aeromonas* spp.	2	(1)	1	(2)	0.80	(0.07–8.96)	1.00
*Campylobacter* spp.	54	(35)	2	(3)	16.04	(3.77–68.18)	<0.01
*C. jejuni*	45	(29)	1	(2)	24.96	(3.36–185.53)	<0.01
*C. coli*	10	(6)	0		-		0.07
*C. spp*	0		1	(2)	-		0.29
Diarrheagenic *E. coli*	29	(19)	6	(10)	2.15	(0.84–5.47)	0.15
ETEC	17	(11)	1	(2)	7.51	(0.98–57.74)	0.03
LTST	0		1	(2)	-		0.29
ST	17	(11)	0		-		<0.01
EPEC	11	(7)	4	(6)	1.11	(0.34–3.62)	1.00
EAEC	4	(3)	1	(2)	1.62	(0.18–14.75)	1.00
*P. shigelloides*	21	(14)	1	(2)	9.56	(1.26–72.70)	0.01
Non-typhoidal *Salmonella* spp.	36	(23)	10	(16)	1.57	(0.73–3.41)	0.28
*V. parahaemolyticus*	7	(5)	1	(2)	2.89	(0.35–23.95)	0.45
Adenovirus	1	(0.6)	0		-		1.00
No Enteric Pathogen Identified	45	(29)	47	(76)	0.13	(0.07–0.26)	<0.01
>1 Enteric Pathogen Detected	36	(23)	5	(8)	3.45	(1.29–9.26)	0.01

^a^Fisher’s exact

Other potential etiologic agents of diarrhea were found infrequently. Seven of eight *Vibrio parahaemolyticus* and two of three *Aeromonas* spp. were isolated from cases. *Shigella, Giardia, Cryptosporidium*, rotavirus or astrovirus were not identified in either cases or controls during the the three study years. Adenovirus was detected in one case specimen from year 2004.

The odds of isolating any enteric pathogen were significantly greater for cases than controls (*p* < 0.01) and significantly more cases than controls provided specimens from which multiple pathogens could be detected (*p* = 0.01) (Table 2). Nearly half (22 of 54) of case specimens positive for *Campylobacter* were also positive for at least one of the following pathogens: adenovirus, diarrheagenic *E. coli*, *P. shigelloides*, or *Salmonella.*

### Antimicrobial Susceptibility testing

Resistance to multiple antibiotics was common among *Campylobacter* isolates found in approximately 95% of both *C. jejuni* and *C. coli* isolates. Resistance to fluoroquinolones, ampicillin and trimethoprim/sulfamethoxazole was similar between the *C. jejuni* and *C. coli* isolates. Fluoroquinolone resistance was very common among the 57 *Campylobacter* isolates: 54 were resistant to nalidixic acid (95%) and 51 (89%) were resistant to ciprofloxacin (Table 3). One *C. coli* isolate was resistant to azithromycin.

**Table 3:** Number and percent of enteric pathogen isolates resistant to selected antibiotics^a^

Pathogen	Number of isolates	Percent resistance						
		AM	AZM	CIP	NA	SXT	TE	≥2 antibiotics
*Aeromonas* spp.	3	100	0	0	67	0	67	67
*Campylobacter* spp.	57	32	2	89	95	19	68	95
*C. jejuni*	47	34	0	89	94	19	68	96
*C. coli*	10	20	10	90	100	20	70	90
Diarrheagenic *E. coli*	40	65	10	0	18	60	63	65
ETEC	19	42	0	0	5	37	37	42
EPEC	16	81	13	0	38	75	81	81
EAEC	5	100	40	0	0	100	100	100
*P. shigelloides*	22	50	0	0	27	27	68	64
Non-typhoidal *Salmonella* spp.	54	33	6	0	35	24	63	48
*V. parahaemolyticus*	8	100	0	0	0	0	0	0

*AM* ampicillin, *AZM* azithromycin, *CIP* ciprofloxacin, *NA* nalidixic acid, *SXT* trimethoprim/sulfamethoxazole, *TE* tetracycline

^a^Multiple species isolates from individual subjects with differing resistance patterns counted as separate isolates

All bacterial species except for *Campylobacter* were susceptible to ciprofloxacin. Regarding diarrheagenic *E.coli*, all EAEC isolates were completely resistant to ampicillin, tetracycline and trimethoprim/sulfamethoxazole. A greater percentage of EPEC isolates were resistant to ampicillin, tetracycline, trimethoprim/sulfamethoxazole and nalidixic acid than ETEC (81 vs 42%, 81 vs 37%, 75 vs 37%, 38% vs 5%, respectively). Resistance to azithromycin was seen only among EAEC (40%) and EPEC isolates (13%). All eight *V. parahaemolyticus* isolates were resistant to ampicillin and had no other antibiotic resistance.

### Questionnaire data

Cases who were positive for *Campylobacter* were more likely to self-report fever, muscle aches (*p* < 0.01) and headache (*p* = 0.02) than cases negative for *Campylobacter* isolation (Table 4). However, after adjustment by logistic regression, only fever and headache remained significantly associated with *Campylobacter* isolation in cases (Table 4). Those reporting abdominal cramps, nausea and vomiting were not significantly more likely to be *Campylobacter* positive. There were no other statistically significant associations between any of the enteric pathogens detected and self-reported symptoms.

**Table 4:** Association between Campylobacter isolation and self-reported symptoms among diarrhea cases

Symptom	Campylobacter isolated n (%) *N* = 54		Campylobacter not isolated n (%) *N* = 101		Odds Ratio (95% CI)		*p*-value	Adjusted Odds Ratio (95% CI)*		*p*-value
Abdominal cramps	41	(76)	73	(72)	1.21	(0.57, 2.59)	0.70			
Fever	40	(74)	24	(24)	9.17	(4.28, 19.64)	<0.01	9.96	(4.50, 22.00)	<0.01
Nausea	36/53	(68)	53	(52)	1.92	(0.96, 3.85)	0.09			
Vomiting	19	(35)	23	(23)	1.84	(0.89, 3.81)	0.13			
Muscle aches	31	(57)	32	(32)	2.91	(1.47, 5.76)	<0.01			
Headache	5	(9)	1	(1)	10.20	(1.16, 89.73)	0.02	15.41	(1.48,160.4)	0.02

*Final model from multivariate logistic regression removing least significant variable stepwise until *p* < 0.1 for all retained variables

A comparison of traveler’s diarrhea risk factors; high-risk foods, source of foods, and previous travels to Cobra Gold, Thailand or Southeast Asia showed no statistically significant difference between cases and controls. Among cases, positivity for *Campylobacter* was not statistically significantly associated with previous travel to Thailand, Southeast Asia, any other overseas location, or previous participation in Cobra Gold exercises (data not shown). Based on responses to the 2002 and 2003 questionnaires, there were no significant associations with all foods and food sources identified (data not shown).

## Discussion

This study focused on determining the diarrhea etiology and antimicrobial susceptibility pattern among US military deployed to Thailand 2002–2004. Despite the differing locations and timeframes studied, *Campylobacter* spp. predominated and was identified in 35% of diarrhea cases, followed by non-typhoidal *Salmonella* spp., diarrheagenic *E.coli*, *P. shigelloides*, *V. parahaemolyticus* and *Aeromonas* spp. While other pathogens were identified in both cases and controls, the isolation of *C. jejuni*, ST-ETEC and *P. shigelloides* was significantly associated with symptomatic infections. Similar prior studies [5, 13–15], and a systematic review of travelers’ diarrhea etiology conducted during 1990 and 2005 reported a prevalence of *Campylobacter* of 23.9% for Southeast Asia [26]. In a recent diarrheal etiology study of a US military exercise in the Philippines in 2014 showed 4 of 7 diarrhea stool samples (57%) were positive for *Campylobacter* spp. [27]. *Campylobacter* continues to be the leading cause of diarrhea in US military personnel deployed to Thailand or Southeast Asia.

Fluoroquinolone resistance among *Campylobacter* isolated from this population is high but consistent with the trend of increasing prevalence over the preceding 15 years: 76% in 1994, 84% in 1995, and 96% in 1998 in *Campylobacter* isolated from similar populations [8, 10]. Additionally, two hospital based traveler’s diarrhea studies in Bangkok, Thailand found *Campylobacter* resistance to ciprofloxacin and nalidixic acid in 83% and 89% of isolates in 2001–2003 [28], and in 91% and 93% of isolates in 2012–2014, respectively [Unpublished data].

Interestingly, none of the other diarrheal pathogens isolated during this study were resistant to ciprofloxacin, despite a moderate frequency of nalidixic acid resistance. The observed level of resistance to nalidixic acid in non-typhoidal *Salmonella* isolates in this study is congruent with a slow but steady increase in prevalence previously reported of 1% in 1991–1992, 4% in 1993–94, 9% in 1995 [12], 21% in 1996–1999 [29], and finally to 35% in 2002–2004 in this study.

The majority of the diarrheal pathogens isolated in this study showed a moderate frequency of resistance to ampicillin, trimethoprim/sulfamethoxazole and tetracycline, but a low frequency of resistance to azithromycin. The level of resistance to azithromycin seen in the *Campylobacter* isolates is consistent with previous reports [12]. Only one *C. coli* isolate was resistant to azithromycin, however, fewer than 20% of all *Campylobacter* isolates were identified as *C. coli*. Fortunately, this organism is still a relatively infrequent cause of acute diarrheal illness. All *V. parahaemolyticus* isolates were resistant to ampicillin but were otherwise susceptible to the other antibiotics tested. Among the antibiotics tested, azithromycin had the broadest spectrum of activity against the pathogens isolated in this study.

However, for diarrhea caused by *Campylobacter* spp., azithromycin was the best available therapeutic agent due to the high level of fluoroquinolone resistance. The American College of Gastroenterology (ACG) Clinical Guideline currently recommends azithromycin for empiric use as first line therapy to treat travelers’ diarrhea in Southeast Asia [30]. Since the prevalence of resistance to azithromycin was low in the other isolated pathogens, treatment with azithromycin would still be effective, even in non-*Campylobacter* diarrheas accompanied by fever or in mixed infections. Continued close monitoring of resistance to azithromycin is essential to maintain effective treatment guidelines.

Limitations of this study result include observation design without an a priori sample size calculation. As the control subjects were selected by cases to have similar exposures, the limited number of enrolled controls may not be truly representative of the population at risk. In addition, controls could potentially develop diarrhea within 2 to 3 days of enrolment weakening the association between specific pathogens and diarrhea. In this study, we also did not test for Norovirus an important cause of travelers’ diarrhea. Despite these limitations and the age of the data, the information on diarrhea etiology and antimicrobial susceptibility as well as disease association remain useful.

## Conclusions

The frequency and severity of traveler’s diarrhea due to *Campylobacter*, coupled with its high rate of fluoroquinolone resistance, are important considerations for clinicians providing advice regarding antibacterial prophylaxis and treatment for civilian and military travelers. The low level of azithromycin resistance observed in this study supports its empiric use for treatment of diarrhea cases among travelers from the US to Thailand.

## Abbreviations

CF: Colonization factor
EAEC: Enteroaggregative *E.coli*
EPEC: Enteropathogenic *E. coli*
ETEC: Enterotoxigenic *E. coli*
LT: Heat-labile enterotoxin
MAC: MacConkey
MSRV: Modified semi-solid Rappaport-Vassiliadis
ST: Heat-stable enterotoxin
